# Upregulation of deubiquitinase PSMD14 in lung adenocarcinoma (LUAD) and its prognostic significance

**DOI:** 10.7150/jca.39539

**Published:** 2020-03-04

**Authors:** Ling Zhang, Hui Xu, Chunping Ma, Jieru Zhang, Yuanjie Zhao, Xiaomei Yang, Shusheng Wang, Dawei Li

**Affiliations:** 1 Center for Translational Medicine, The Affiliated Zhangjiagang Hospital of Soochow University, 68 Jiyang W Rd, Suzhou, 215600, China.; 2 Department of Thoracic Surgery, The Affiliated Zhangjiagang Hospital of Soochow University, 68 Jiyang W Rd, Suzhou, 215600, China.; 3 Department of Respiratory & Critical Care Medicine, The Affiliated Zhangjiagang Hospital of Soochow University, 68 Jiyang W Rd, Suzhou, 215600, China.; 4 Department of General Surgery, The Affiliated Zhangjiagang Hospital of Soochow University, 68 Jiyang W Rd, Suzhou, 215600, China.; 5 Department of Emergency, The Affiliated Zhangjiagang Hospital of Soochow University, 68 Jiyang W Rd, Suzhou, 215600, China.

**Keywords:** PMSD14, deubiquitinating enzyme, lung adenocarcinoma, prognosis, senescence, apoptosis

## Abstract

PSMD14 is a 19S-proteasome-associated deubiquitinating enzyme that facilitates protein degradation by the 20S proteasome core particle. Although accumulating evidence indicates that PSMD14 has emerged as a critical oncogenic factor by promoting tumor growth, the expression and function of PSMD14 in non-small cell lung cancer (NSCLC) remain largely unknown. In this study, we assessed PSMD14 expression and correlated it with clinical-pathological features and patient survival in NSCLC. We also determined the roles of PSMD14 in the regulation of lung adenocarcinoma (LUAD) cell growth. The results showed that PSMD14 expression was significantly upregulated in human NSCLC tissues compared with adjacent non-cancerous tissues. The PSMD14 level was associated with tumor size, lymph node invasion, and TNM stage in LUAD patients. Importantly, high PSMD14 expression was associated with poor overall survival (OS) and disease-free survival (DFS) in LUAD patients. Further, knockdown of PSMD14 significantly inhibited cell growth and caused G1 arrest and cellular senescence by increasing p21 stability in LUAD cells. PSMD14 knockdown also promoted cell apoptosis by increasing cleaved caspase-3 levels in H1299 cells. PSMD14 may serve as a potential prognostic marker and therapeutic target for LUAD patients.

## Introduction

Lung cancer has the highest morbidity and mortality rates of all cancers 1. Non-small cell lung cancer (NSCLC) comprises approximately 85% of all lung cancers and includes adenocarcinoma, squamous-cell carcinoma, and large-cell carcinoma 2. Although malignant proliferation of lung epithelial cells is a common feature of NSCLC, its pathogenesis has not been fully elucidated 2. Currently, the main treatments for NSCLC are surgery, radiotherapy, and drug therapy. For terminal patients, drug treatment is not effective 3, 4. There is an urgent need to identify prognostic markers and effective drug targets to improve patient survival. An in-depth understanding of the pathogenesis of NSCLC is crucial for identifying potential therapeutic targets and effectively treating the disease.

PSMD14, also known as Rpn11/POH1, is a deubiquitinating enzyme within the 19S proteasomal subunit responsible for substrate deubiquitination during proteasome degradation 5-9. PSMD14 plays a critical role in cell growth, senescence, and apoptosis 10-12. PSMD14 is involved in a series of important physiological and pathological processes such as DNA damage repair 13-16, tumor formation 17-19, and inflammation 20. PSMD14 promotes cell growth and proliferation of gliomas, colon cancer, liver cancer, esophageal cancer, and breast cancer, and enhances the resistance of tumor cells to radiation and chemotherapy drugs 17-23. Reduced PSMD14 expression inhibits tumor growth by inducing cell cycle arrest, senescence, or apoptosis. Previous studies have demonstrated that PSMD14 promotes tumor progression by regulating key anti-cancer proteins such as c-jun, p53, SNAIL, mTOR, and E2F1 17-22, 24. Importantly, small molecule inhibitors targeting PSMD14 were shown to be prospects for potential clinical application 25-28. However, the expression of PSMD14 in NSCLC and the underlying mechanism by which PSMD14 is involved in NSCLC development remain largely unknown.

In this study, we report that the expression of the deubiquitinase PSMD14 is significantly upregulated in NSCLC tissues. We analyzed the relationship between the PSMD14 expression level and clinical-pathological characteristics. High PSMD14 expression predicted a poor overall survival (OS) and disease-free survival (DFS) in lung adenocarcinoma (LUAD) patients. Furthermore, the biological functions of PSMD14 were examined using LUAD cells. Our results indicate that PSMD14 exerted an oncogenic role in LUAD.

## Materials and methods

### Patients

We assessed PSMD14 expression in 17 NSCLC patients. The patients who underwent curative surgery at The Affiliated Zhangjiagang Hospital of Soochow University from March to July 2019 were enrolled in this study. Histological confirmation of primary NSCLC was obtained from the Department of Pathology at the hospital. None of the patients received preoperative adjuvant chemotherapy. The tumor stage at the time of diagnosis was assessed according to the American Joint Committee on Cancer guidelines (http://www.cancerstaging.org/). For the NSCLC specimens, matched non-cancerous lung tissues were sampled from the resection margins far from the originally located tumor site. The tissue samples were immediately stored at -80°C after surgery for later mRNA isolation, protein extraction, or immunostaining. The demographical and clinical features of all NSCLC patients are shown in Supplementary Table S1. Written permission was requested and received from all NSCLC patients in the study. The use of human specimens was approved by The Zhangjiagang Hospital Institutional Review Board (No.2019001).

### Bioinformatics analysis

To determine PSMD14 expression in NSCLC patients, we also analyzed and compared the data from Gene Expression Omnibus (GEO; https://www.ncbi.nlm.nih.gov/geoprofiles/17271077) under the following accession numbers: human adjacent normal lung (GSM47958 to GSM47976) and LUAD (GSM36757 to GSM36776) 29. To understand the correlation between PSMD14 levels and clinical-pathological characteristics and NSCLC patient overall survival, we made use of the publicly available clinical information provided by cBioportal for The Cancer Genome Atlas (TCGA) databases (http://www.cbioportal.org/) 30, 31, which included basic demographic and clinical information as well as the survival status following surgery. We retrieved clinical information from NSCLC patients, including 518 patients with LUAD and 484 patients with lung squamous cell carcinoma (LUSC), for correlation analysis. We extracted the tumor cohorts from 509 LUAD patients and 478 LUSC patients, stratifying by higher and lower PSMD14 expression for OS analysis. Data from 420 LUAD patients and 364 LUSC patients were used in DFS analysis. We also retrieved data from 388 LUAD patients with complete demographic and clinical information for OS and DFS for multivariate regression analysis.

### Cell culture

Human NSCLC cell lines, H1299 and A549, were cultured in Dulbecco's Modified Eagle Medium (DMEM) supplemented with 10% fetal bovine serum (FBS) and antibiotics (100 U/ml penicillin and 100 µg/ml streptomycin). All cells were cultured at 37°C in a humidified incubator with 5% CO_2_.

### Real-time PCR (RT-PCR)

Total RNA was isolated from tissues or cultured cells using Trizol reagent (Invitrogen, Carlsbad, CA, USA) following the manufacturer's protocol and RNA was reverse transcribed into cDNA using the RevertAid H Minus First Strand cDNA Synthesis Kit (K1632, Thermo Fisher Scientific, Waltham, MA, USA). RT-PCR was performed in triplicate using a SYBR green mix (Applied Biosystems, Foster City, CA, USA) and a QuantStudio Dx Real-Time PCR Instrument (Applied Biosystems) under the following conditions: 10 min at 95°C, followed by 40 cycles of 95°C for 15 s and 60°C for 1 min. GAPDH was used as an internal reference for normalization. The primer sequences (Sangon, Shanghai, China) were as follows: PSMD14, F: 5'-GTCAGGAACAGGTGTCAGTGT-3', R: 5'-AACCAACAACCATCTCCGGC-3'; GAPDH, F: 5'-CAGGAGGCATTGCTGATGAT-3', R: 5'-GAAGGCTGGGGCTCATTT-3'; P21, F: 5'-CCATGTGGACCTGTCACTGTCTT-3', R: 5'-CGGCCTCTTGGAGAAGATCAGCCG-3'; and PUMA, F: 5'-GGTCCTCAGCCCTCGCTCTC-3', R: 5'-CTTGTCTCCGCCGCTCGTAC-3'.

### Western blotting and antibodies

The tissues and cells were collected and homogenized in lysis buffer (P0013, Beyotime, Shanghai, China) containing protease inhibitor cocktail (P8340, Sigma, St Louis, Missouri, USA). The proteins were extracted and the concentration was measured using a BCA protein assay kit (23227, Pierce, Rockford, IL, USA). Equal quantities of protein from each sample were loaded for SDS-PAGE. After electrophoresis, the proteins were transferred onto nitrocellulose membranes (GE Healthcare, Munich, Germany). The membranes were blocked in 5% nonfat milk for 1 h at room temperature (RT) and subsequently incubated overnight at 4°C with the following primary antibodies: PSMD14 (4197, Cell Signaling Technology, Danvers, MA, USA), p53 (sc-126, Santa Cruz Biotechnology, CA, USA), p21 (sc-53870, Santa Cruz), PUMA (sc-377015, Santa Cruz), caspase-3 (19677, Proteintech, Rosemont, IL, USA), cleaved caspase-3 (9661, Cell Signaling Technology, Danvers, MA, USA), and GAPDH (G9545, Sigma). After extensive washes, the membranes were incubated with the appropriate secondary antibodies for 1 h at RT. The target band signals were developed using an enhanced chemiluminescence solution (ECL, WBKLS0500, Millipore, Bedford, MA, USA) and ChemiDoc XRS (Bio-Rad, CA, USA) detection system. The signals were quantified using ImageJ software (National Institutes of Health, Bethesda, MD, USA).

### Immunohistochemistry (IHC)

Frozen sections with a thickness of 6 µm were fixed in 4% formaldehyde for 15 min at RT. The sections were washed twice in phosphate-buffered saline (PBS) containing 0.3% Triton X-100 for 5 min each and then blocked with 10% normal goat serum for 1 h at RT. The sections were incubated with PSMD14 antibody (4197, Cell Signaling Technology) at 1:100 overnight at 4°C and further treated with 0.3% H_2_O_2_ for 15 min. After extensive washes, the biotin-conjugated secondary antibody was added to the sections, which were further incubated for 1 h at RT. After more washes, a solution containing streptavidin conjugated HRP (Beyotime, China) was added to the sections. The sections were incubated for 1 h at RT and then washed three times in PBS for 5 min each. The slides were developed with 3'-diaminobenzidine (DAB, Beyotime, China) for 10 min at RT, followed by rinsing in H_2_O for 5 min. The slides were counterstained in hematoxylin, dehydrated, cleared, and mounted. Immunohistochemical staining was observed using a Leica upright microscope (DM4000B, Leica Microsystems, Heidelberg, Germany).

### RNA interference

H1299 and A549 cells were transfected with specific siRNAs using Lipofectamine 2000 (Invitrogen, USA) according to the manufacturer's protocol. Two individual PSMD14 siRNAs (siPSMD14-1 and siPSMD14-2, reference 12) and scrambled negative control siRNA (NC) were synthesized by Genepharma (Shanghai, China). The nucleotide sequences of the siRNAs were as follows: siPSMD14-1: 5'-GUACUUAUGACCUCAAAUA-3'; siPSMD14-2: 5'-CAGAAGAUGUUGCUAAAAUU-3'; and NC: 5'-UUCUCCGAACGUGUCACGUTT-3'.

### Cell viability and proliferation assays

Cell viability was detected using the Cell Counting Kit-8 (CCK-8, CK04, Dojindo, Kumamoto, Japan). H1299 and A549 cells were cultured in 96-well plates. Cell viability was monitored 1, 3, and 5 days after being transfected with scrambled or PSMD14 siRNAs, following the manufacture's protocol. CCK-8 optical density (OD) at 450 nm was measured using the Varioskan Flash multimode reader (Thermo Fisher Scientific). For the crystal violet assay to determine the viability of cultured cells, 5x10^4^ cells were plated in each well of 6-well plates. The cells were fixed with methanol and stained with 0.1% crystal violet 72 hours after transfection with NC or si-PSMD14.

### Flow cytometry analysis

H1299 and A549 cells transfected with NC or si-PSMD14 were harvested 72 hours after transfection. After staining with propidium iodide (PI) using the PI/RNase Staining Buffer (BD Biosciences, San Diego, CA, USA) by following the manufacturer's protocol, the cells were analyzed with a Navios Flow Cytometer (Beckman Coulter, Brea, CA, USA).

### Senescence-associated β-galactosidase (SA-β-Gal) staining

SA-β-Gal staining was performed after H1299 and A549 cells were transfected with NC or si-PSMD14 for 72 hours. The Senescent Cells Staining Kit (C0602, Beyotime) was used according to the manufacturer's instructions. The cells were viewed under an inverted fluorescent microscope (DMI4000B, Leica Microsystems). Representative pictures were imaged for each condition. The number of SA-β-Gal-positive cells was counted and expressed as a percentage of the total number of cells in five separate fields. The means of SA-β-Gal-positive cells and standard deviation were derived from two independent experiments.

### Protein stability

A549 cells were transfected with NC or si-PSMD14 for 48 h and then treated with 50 µg/ml cycloheximide (CHX) for various periods to block protein synthesis. Crude extracts were prepared at indicated times and the protein levels of p53, p21, and PUMA were assessed using western blot analysis.

### Statistical analysis

All data are presented as mean ± SD. GraphPad InStat software (GraphPad Prism 5.01, GraphPad Software Inc., San Diego, CA) and SPSS Statistics 17.0 were used for statistical analysis. The results were analyzed a using student's t-test for comparisons of two groups or one-way analysis of variance (ANOVA) for comparisons of multiple groups. The relationships between PSMD14 expression and clinical-pathological parameters were analyzed by the Chi-square or Fisher's exact test. Survival curves were plotted using the Kaplan-Meier method and compared by the log-rank test. Univariate and multivariate Cox regression analyses were used to assess variables associated with OS and DFS in LUAD patients. Statistical significance was accepted at *p* < 0.05.

## Results

### PSMD14 expression is upregulated in human NSCLC tissues

**Figure 1:**
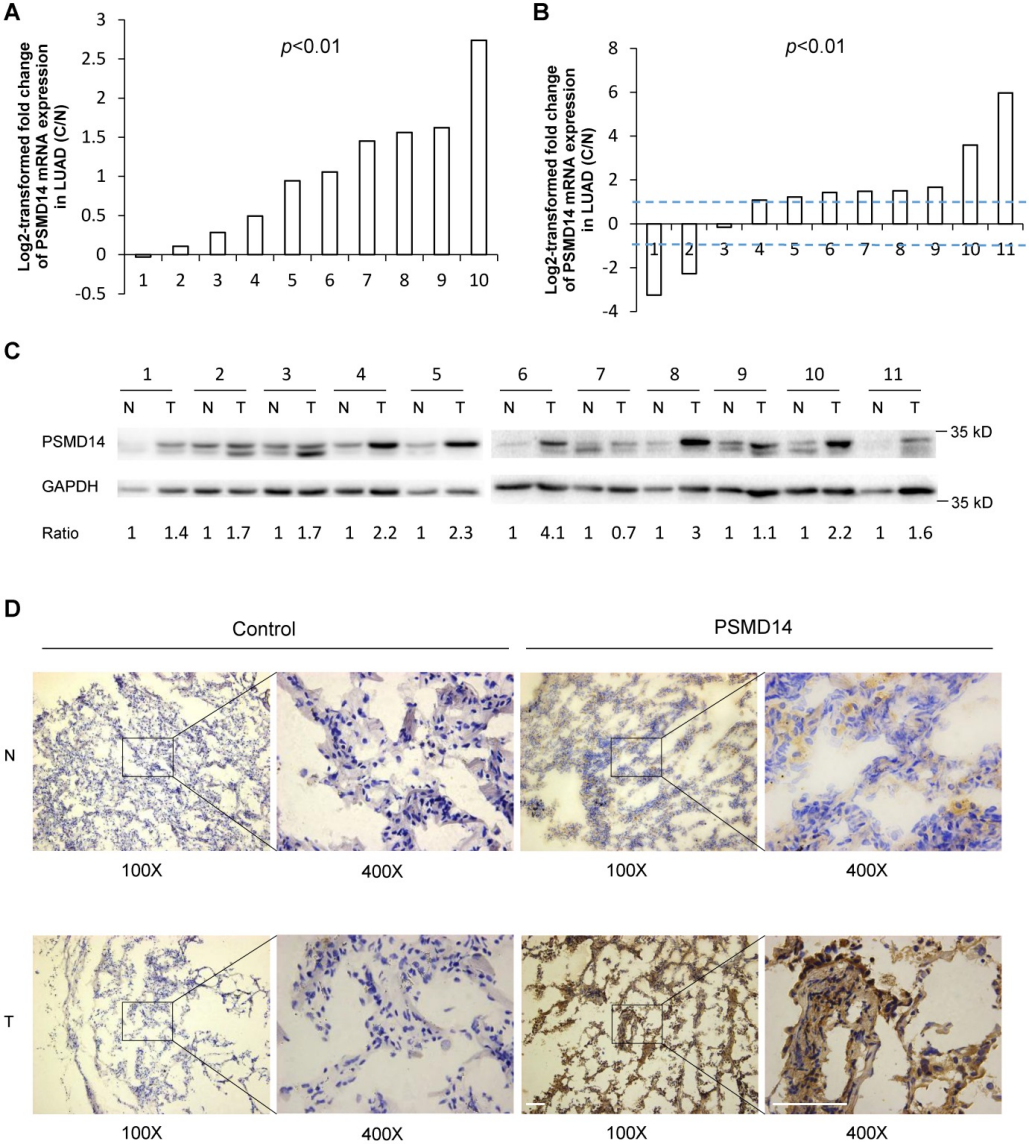
PSMD14 expression is upregulated in LUAD tissues. (A) Relative mRNA expression of PSMD14 was determined by tissue microarray in 10 pairs of LUAD tumor tissues and adjacent non-cancerous tissues. The PSMD14 expression was retrieved from the GEO databases and re-analyzed. (B) Relative mRNA expression of PSMD14 in 11 pairs of LUAD tumor tissues and adjacent non-cancerous control tissues was determined by RT-PCR. The ratio of PSMD14 expression in tumor tissues to controls for each patient is shown as a log2-transformed fold change. A two-fold increase or decrease was arbitrarily set as cut-off values. (C) PSMD14 expression in 11 paired tumor and adjacent non-cancer tissues from LUAD patients was determined by western blotting. GAPDH was used as an internal control. (D) Typical IHC staining of PSMD14 in the paired tumor and adjacent non-cancerous tissues. The staining was performed in two pairs of tissues and a representative photograph was shown. The bar represents 100 µm. LUAD, lung adenocarcinoma; N, normal tissue; T, tumor tissue.

PSMD14, a proteasome-associated deubiquitinase, has emerged as an oncogenic factor by promoting tumor cell growth 17-19, 21, 22. To clarify how PSMD14 functions in NSCLC pathogenesis, we first analyzed the expression of PSMD14 in human LUAD tissues using raw microarray data that was downloaded from GEO. We found that PSMD14 expression was significantly upregulated in LUAD tissues compared to the corresponding adjacent non-tumor tissues (Fig. 1A). Then, RT-PCR and western blot analysis were used to investigate the expression of PSMD14 in 11 paired tumor and adjacent non-cancer control tissues from LUAD patients. The results showed that both the mRNA and protein levels of PSMD14 were markedly increased in tumor tissues compared to the controls (Fig. 1B and C). While the PSMD14 mRNA level was not significantly higher in LUSC tissues, the PSMD14 protein levels were significantly elevated (Fig. S1A and S1B). We also performed immunohistochemical staining to detect PSMD14 in tumor tissues and controls from LAUD patients. PSMD14 staining was primarily detected in the cytoplasm of normal cells whereas its staining was detected in both the cytoplasm and nuclei of tumor cells. The staining showed higher PSMD14 levels in tumors compared to the non-cancerous tissues (Fig. 1D and Fig. S2).

### PSMD14 expression is associated with clinical features in LUAD patients

The relationships between PSMD14 expression and demographic and clinical features of NSCLC patients were assessed using the TCGA databases. PSMD14 expression was significantly associated with tumor size, lymph node invasion, and TNM stage in LUAD patients (Table 1). The PSMD14 level was positively associated with a history of smoking and it was marginally significant with tumor size of LUSC patients. Expression was not significantly correlated with the other clinical or pathological characteristics of LUSC patients (Table S2). Taken together, these results suggest that PSMD14 levels are specifically associated with LUAD severity and progression.

### High PSMD14 expression predicts a poor prognosis in LUAD patients

**Figure 2:**
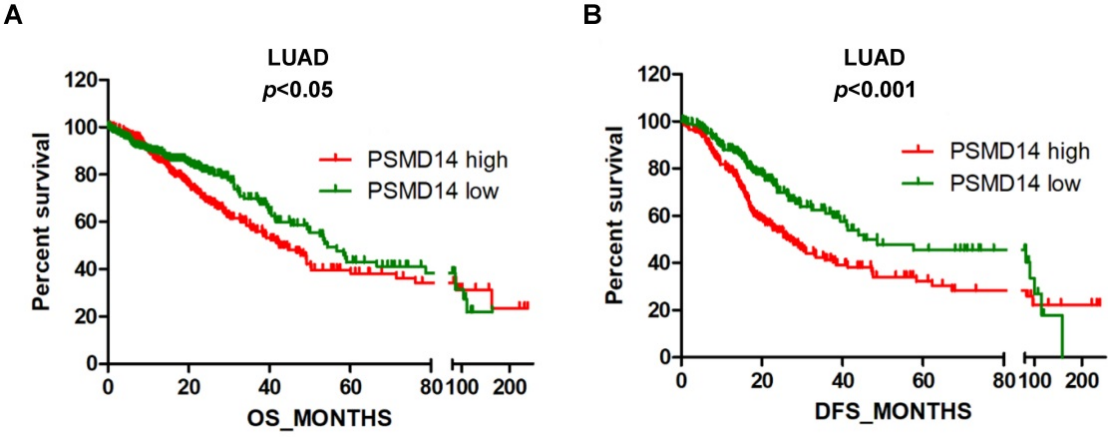
High PSMD14 expression indicates a poor OS and DFS in LUAD patients. (A-B) Kaplan-Meier survival curves of higher and lower PSMD14 expression for OS (n=509) and DFS (n=420) in LUAD patients. The patients' information was retrieved from the cBioportal for the Cancer Genome Atlas (TCGA) databases and analyzed by a log-rank test. LUAD, lung adenocarcinoma; OS, overall survival; DFS, disease-free survival.

Next, we assessed the prognostic significance of PSMD14 expression in NSCLC patients through mining in the TCGA databases. LUAD patients with reduced PSMD14 expression had significantly longer OS and DFS compared to patients with higher transcript levels (Fig. 2A and B). The median for OS and DFS were 42.5 and 26.9 months for patients with higher PSMD14 expression and 54.3 and 45.3 months for patients with lower PSMD14 levels, respectively. However, PSMD14 expression was not significantly correlated with prognosis in LUSC patients (Fig. S3A and S3B). Univariate Cox regression analysis demonstrated that tumor size, lymph node metastasis, TNM stage, and PSMD14 expression were correlated with OS and DFS in LAUD patients. Multivariate Cox regression analysis, including the above factors, revealed that lymph node metastasis was an independent prognostic factor for OS and tumor size was a prognostic factor for DFS in LAUD patients (Table 2).

### PSMD14 knockdown induces cell cycle arrest, apoptosis, and senescence in LUAD cells

**Figure 3:**
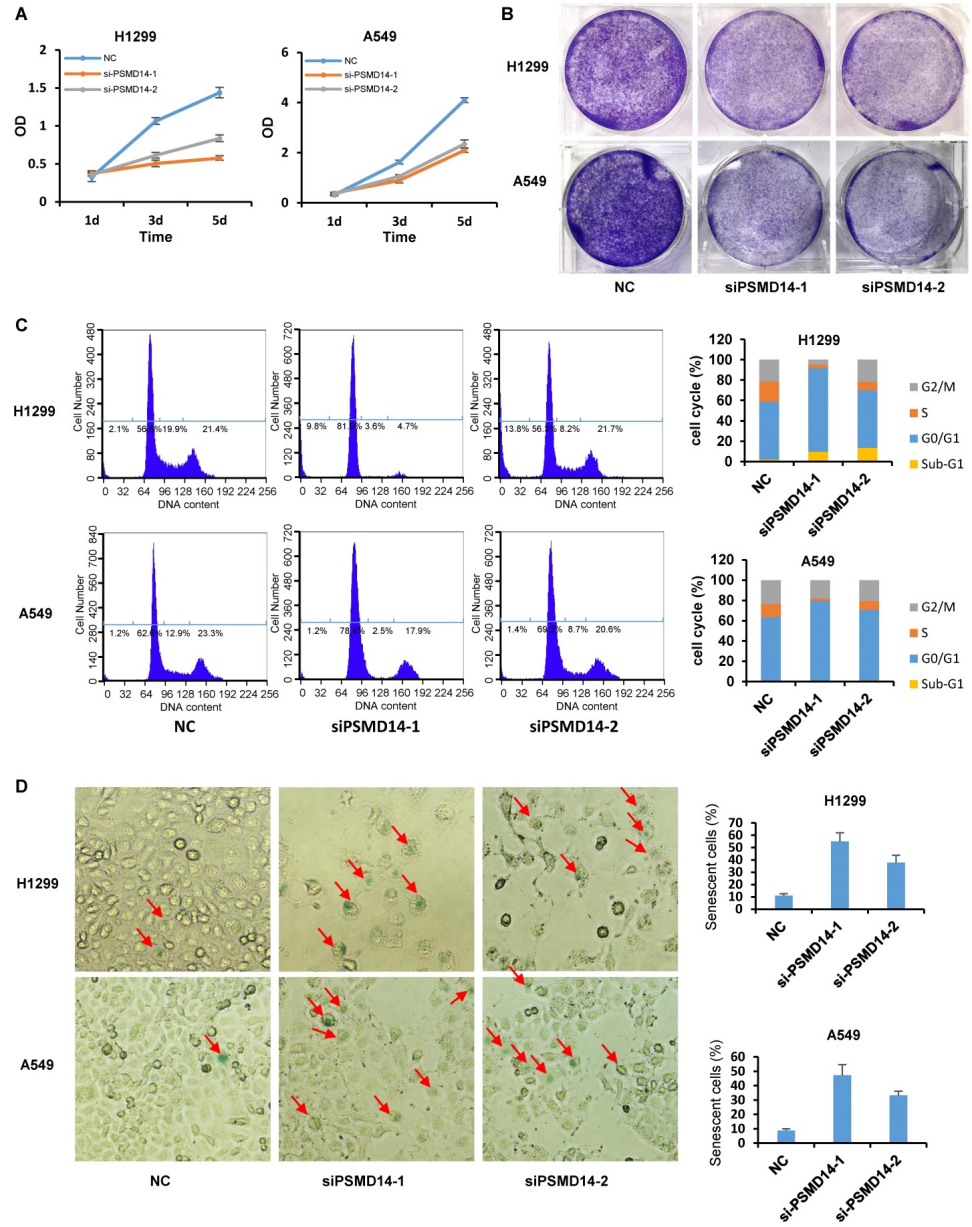
PSMD14 knockdown induces cell cycle arrest, apoptosis, and senescence in LUAD cells. (A) The viability of H1299 and A549 cells transfected with NC, si-PSMD14-1, and si-PSMD14-2 was assessed by CCK8 assays. The OD value was determined 1, 3, and 5 days post-transfection. The assays were performed using four replicates for each group. The experiments were repeated twice and a representative result is shown. (B) Crystal violet staining was performed to assess the survival of H1299 and A549 cells treated with NC, si-PSMD14-1, and si-PSMD14-2. The experiments were repeated twice and a representative staining is shown. (C) H1299 and A549 cells were transfected with NC, si-PSMD14-1, and si-PSMD14-2 for 72 hours. Cell cycle and apoptosis were determined by PI staining followed by flow cytometry analysis (Left panel). The percentage in each phase of the cell cycle was also demonstrated (Right panel). The experiments were repeated twice and a representative result was shown. (D) PSMD14 knockdown induced cellular senescence in H1299 and A549 cells. Exponentially growing H1299 and A549 cells were transfected with NC, si-PSMD14-1, and si-PSMD14-2. After 72 hours, the cells were fixed and incubated with SA-β-Gal overnight. The senescence-like phenotype was imaged with microscopy (10×). The number of SA-β-Gal-positive cells was counted and expressed as a percentage of the total number of cells in five separate fields. The means of SA-β-Gal-positive cells and standard deviations were derived from two independent experiments. NC, scrambled control siRNA; PI, propidium iodide; SA-β-Gal, senescence-associated β-galactosidase.

**Figure 4:**
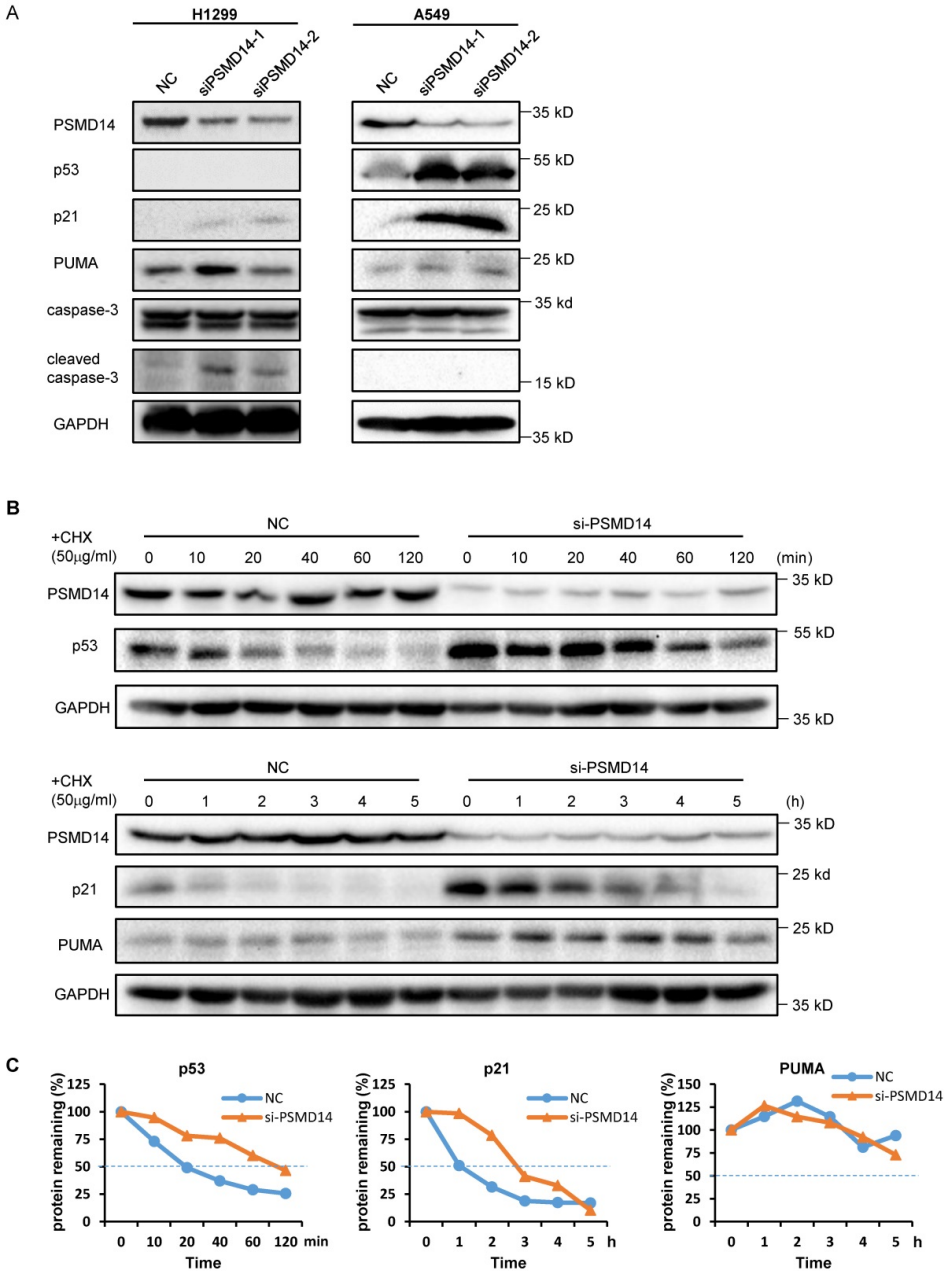
Knockdown of PSMD14 increased the stability of p53 and p21 in LUAD cells. (A) The whole-cell lysates from H1299 and A549 cells were extracted after the cells were transfected with NC, si-PSMD14-1, and si-PSMD14-2 for 72 hours. Western blot analysis was performed with the indicated antibodies. (B) A549 cells transfected with NC or si-PSMD14-1 were treated with 50 µg/ml CHX and collected at the indicated time points. Western blot analysis was performed with p53, p21, and PUMA antibodies. GAPDH was used as an internal control. (C) Quantification of the p53, p21 and PUMA levels relative to GAPDH expression is shown. NC, scrambled control siRNA; CHX, cycloheximide.

A previous study by Ann Byrne, et al. 12 indicated that the knockdown of human deubiquitinase PSMD14 induces cell cycle arrest and senescence in human tumor cells. To investigate the role of PSMD14 in NSCLC pathogenesis, we used two LUAD cell lines, H1299 (p53 null) and A549 (p53 wild-type), and transfected them with two different PMSD14 siRNAs. A CCK8 assay (Fig. 3A) and crystal violet staining (Fig. 3B) showed that cell proliferation was significantly inhibited after PSMD14 expression was knocked down in both cell lines. These results suggested that PSMD14 played an essential role in promoting lung cell proliferation. The cell proliferation/viability assays did not distinguish whether reduced cell numbers were caused by cell cycle arrest or by cell death (cytotoxicity). To better understand the effect of PSMD14 knockdown on decreased cell viability, we examined cell cycle and apoptosis by PI staining followed by flow cytometry analysis. PSMD14 knockdown resulted in G1 phase arrest and decreased S phase in both H1299 and A549 cells. Moreover, PSMD14 knockdown induced significant cell apoptosis (sub-G1 peak) in H1299 cells but not in A549 cells (Fig. 3C). Senescence appears to act as a barrier to prevent cells from undergoing proliferation 32. Next, we assessed cell senescence after PSMD14 was knocked down from LUAD cells. We found cell morphology changed from spindle shape to an enlarged, flattened and irregular shape after PSMD14 was knocked down from H1299 and A549 cells. SA-β-Gal staining was conducted to evaluate cellular senescence after the PSMD14 knockdown. We found that SA-β-Gal-positive cells were significantly increased in H1299 and A549 cells after PSMD14 expression was depleted in these cells (Fig. 3D). These results indicated that knockdown of PSMD14 significantly enhanced cellular senescence. Consistently, knockdown of PSMD14 expression significantly increased p21 levels in both cell lines and also increased cleaved caspase-3 levels in H1299 cells. We also observed that p53 and PUMA levels were increased in A549 cells whereas caspase-3 expression remained unchanged following PSMD14 knockdown in both cell lines (Fig. 4A). To investigate whether the knockdown of PSMD14 affects protein stability, we examined the half-life of p53, p21, and PUMA after the cells were treated with CHX to block protein synthesis. The results demonstrated that PSMD14 levels remained constant even after A549 cells were treated with CHX for 5 hours. Importantly, p53 and p21 protein stabilities were significantly increased after PSMD14 was knocked down in A549 cells (Fig. 4A-C). We did not observe an obvious change in the stability of the PUMA protein (Fig. 4B and C). The mRNA levels of p21 and PUMA were not significantly higher (Fig. S4), supporting the idea that PSMD14 may regulate p21 protein stability in a post-transcriptional manner.

## Discussion

Lung cancer has the highest mortality rate of all cancers in the world 33. It often metastasizes before significant symptoms, leading to the low five-year survival rate. Therefore, the diagnosis and treatment of NSCLC are particularly important, urgently requiring sensitive and specific lung cancer molecular markers. PSMD14 is a deubiquitinating enzyme responsible for substrate deubiquitination during proteasomal degradation. Although the role of PSMD14 has been reported in several cancer types 17, 19, 21, 22, 25, 34, its involvement in lung cancer has not been elucidated. In this study, we found that the level of PSMD14 was significantly upregulated in NSCLC tissues, suggesting its tumor-promoting effects in NSCLC. Consistently, high PSMD14 levels were closely related to larger tumor size, lymph node metastases, and advanced TNM stage, with a predicted poor OS and DFS in LUAD patients. Thus, PSMD14 may serve as a potential prognostic marker for NSCLC.

To further investigate the role of PSMD14 in NSCLC, we made use of p53 null H1299 and p53 wild-type A549 LUAD cell lines. We found that PSMD14 knockdown inhibited cell survival by inducing G1 phase cell cycle arrest and cellular senescence in both cell lines. These findings were consistent with the previous report by Ann Byrne et al. 12, which indicated that knockdown of human deubiquitinase PSMD14 induced cell cycle arrest and senescence in human tumor cells. Interestingly, we also detected a significant portion of cells committed to apoptosis after PSMD14 was depleted in H1299 but not A549 cells. We observed cleaved caspase-3 level was increased in H1299 cells. These findings indicated that PSMD14 knockdown suppressed tumor growth by triggering different molecular pathways in the LUAD cells.

Cyclin-dependent kinase (CDK) inhibitor p21 is one of the factors that promotes cell cycle arrest and senescence in response to a variety of stimuli. P21 expression is regulated by both p53-dependent and p53-independent mechanisms 35. We detected a significant increase in p53 levels, concomitantly with an increase in p21 and PUMA levels after PSMD14 depletion in p53 wild-type A549 cells. However, we cannot detect an increase in mRNA levels of p21 and PUMA in these cells, suggesting that the regulation of both proteins was not dependent on p53-mediated transcription. Supportively, we found that p21 was upregulated in both p53 null H1299 and p53 wild-type A549 cells after PSMD14 expression was ablated, suggesting that PSMD14 regulated cell cycle and senescence through a p53-independent pathway. Both p53 and p21 are fast turnover proteins in cells and their degradation is highly dependent on an intact proteasome 36, 37. Proteasome disruption caused by PSMD14 depletion will lead to abnormal accumulation of these proteins.

## Conclusions

In conclusion, our study revealed that PSMD14 was upregulated in NSCLC. The high PSMD14 expression was associated with progressive disease and predicted poor OS and DFS in LUAD patients. Knockdown of PSMD14 inhibited cell proliferation, induced cell cycle arrest and apoptosis, and also resulted in cellular senescence in LUAD cells. PSMD14 may serve as a potential prognostic marker and therapeutic target for NSCLC.

## Supplementary Material

Supplementary figures and tables.

## Figures and Tables

**Table 1:** The expression of PSMD14 was correlated with demographical and clinicopathological characteristics in LUAD patients.

	PSMD14 High	PSMD14 Low	*p* value
**Age**			
≥60	175	187	0.258
<60	74	63	
**Sex**			
Male	124	115	0.428
Female	135	144	
**Tobacco smoking history**			
Stage 3-5	150	159	0.410
Stage 1-2	102	93	
**Other malignancy history**			
Negative	212	214	0.818
Positive	47	45	
**Laterality**			
Left	98	104	0.611
Right	153	148	
**Location of lung parenchyma**			
Peripheral lung	69	58	0.074
Central lung	26	38	
**Residual tumor**			
Negative (R0)	170	176	0.147
Positive (R1/R2)	12	6	
**Tumor Size**			
T1	72	98	0.016*
T2-T4	185	160	
**Lymph node invasion**			
Negative	154	178	0.025*
Positive	99	75	
**Distant metastasis**			
Negative	172	177	0.301
Positive	15	10	
**Tumor TNM stage**			
I/II	188	211	0.014*
III/IV	67	44	

The clinical information from 518 LUAD patients was retrieved from cBioportal for The Cancer Genome Atlas (TCGA) databases for correlation analysis. LUAD: Lung adenocarcinoma; *p<0.05 was considered significant.

**Table 2:** Univariate and multivariate Cox regression analysis of prognostic factors of survival in LUAD patients

Parameters	Overall survival (OS, n=388)			Disease-free survival (DFS, n=388)	
	HR (95% CI)	p value		HR (95% CI)	p value
**Univariate regression analysis**					
Age	0.961 (0.615-1.502)	0.860		1.391 (0.965-2.006)	0.077
Sex	0.909 (0.600-1.378)	0.654		0.972 (0.709-1.332)	0.858
Tobacco smoking	1.305 (0.845-2.017)	0.230		1.342 (0.963-1.868)	0.082
Other malignancy	1.339 (0.765-2.345)	0.307		1.257 (0.838-1.887)	0.269
Tumor size	1.804 (1.124-2.897)	0.015*		1.885 (1.324-2.684)	<0.001*
Lymph node metastasis	2.604 (1.721-3.941)	<0.001*		1.661 (1.203-2.295)	0.002*
TNM stage	2.069 (1.297-3.301)	0.002*		1.435 (0.973-2.117)	0.068
PSMD14 expression	1.218 (1.045-1.419)	0.011*		1.147 (1.015-1.295)	0.028*
**Multivariate regression analysis**					
Tumor size	1.424 (0.873-2.324)	0.157		1.716 (1.194-2.466)	0.004*
Lymph node metastasis	2.105 (1.304-3.399)	0.002*		1.414 (0.971-2.058)	0.070
TNM stage	1.238 (0.729-2.101)	0.430		1.015 (0.652-1.579)	0.949
PSMD14 expression	1.146 (0.978-1.343)	0.091		1.104 (0.972-1.254)	0.127

A total of 388 LUAD patients with complete demographic and clinical information were extracted from the cBioportal for the Cancer Genome Atlas (TCGA) databases for multivariate regression analysis. HR: Hazard ratio, CI: Confidence interval. *p<0.05 was considered significant.

## Abbreviations

LUAD: lung adenocarcinoma
LUSC: lung squamous cell carcinoma
NSCLC: non-small cell lung cancer
OS: overall survival
DFS: disease-free survival
GEO: Gene Expression Omnibus
TCGA: The Cancer Genome Atlas
DMEM: Dulbecco's Modified Eagle Medium
FBS: fetal bovine serum
RT-PCR: Real-time PCR
ECL: enhanced chemiluminescence
IHC: immunohistochemistry
PBS: phosphate-buffered saline
PI: propidium iodide
SA-β-Gal: senescence-associated β-galactosidase
CHX: cycloheximide
