# Supplementary material for: Upregulation of deubiquitinase PSMD14 in lung adenocarcinoma (LUAD) and its prognostic significance
Source: J Cancer. 2020 Mar 4;11(10):2962–71. doi: 10.7150/jca.39539 (PMC7086243; doi:10.7150/jca.39539)
Supplement: Supplementary file 1 — Supplementary figures and tables. [file jcav11p2962s1.pdf]

Supplementary data

Table S1 The demographical and clinical features of NSCLC patients in this study

Patient #	Age	Gender	Histological type	Tobacco smoking history	Other malignancy history	Laterality	Tumor size	Lymph node invasion	Distant metastasis	TNM stage	Other disease history
**1**	69	F	LUAD	No	No	R-Upper	T1	N0	No	Stage IA	No
**2**	80	F	LUAD	No	No	R-Upper	T1	N0	No	Stage IA	No
**3**	79	F	LUAD	No	No	R-Middle	T2	N0	No	Stage IB	No
**4**	71	M	LUSC	Yes	No	R-Upper	T2	N2	No	Stage IIIA	Hypertension
**5**	80	F	LUAD	No	Esophageal Cancer	L-Upper	T2	N0	No	Stage IB	No
**6**	64	F	LUAD	No	Bilateral Malignancy	L-Lower	T1	N0	No	Stage IA	Hypertension
**7**	64	M	LUAD	Yes	No	R-Upper	T2	N1	No	Stage IIB	No
**8**	79	F	LUAD	No	No	R-Lower	T1	N0	No	Stage IA	Hypertension
**9**	66	F	LUAD	No	No	L-Lower	T2	N2	No	Stage IIIA	No
**10**	72	M	LUSC	Yes	No	L-Lower	T4	N0	No	Stage IIIA	Hypertension
**11**	67	F	LUSC	No	No	L-Lower	T1	N0	No	Stage IA	No
**12**	75	F	LUAD	No	No	R-Upper	T2	N0	No	Stage IB	No
**13**	64	M	LUSC	Yes	No	R-Lower	T3	N0	No	Stage IIB	No
**14**	66	F	LUAD	No	No	L-Upper	T1	N0	No	Stage IA	Cerebral infarction; Hypertension
**15**	66	M	LUAD	Yes	No	L-Lower	T2	N0	No	Stage IB	No
**16**	74	M	LUSC	Yes	No	R-Middle	T2	N0	No	Stage IB	No
**17**	56	M	LUSC	Yes	No	L-Upper	T2	N0	No	Stage IB	No

Neoplasm disease stage was determined according to the American Joint Committee on Cancer Code (7th version). NSCLC, Non-small-cell lung carcinoma; LUAD, lung adenocarcinoma; LUSC, Lung squamous cell carcinoma.

Table S2 Correlation between PSMD14 expression and demographical and clinical-pathological features in LUSC patients

	PSMD14 High	PSMD14 Low	*p* value
**Age**			
≥60	188	200	0.185
<60	49	38	
**Sex**			
Male	178	180	0.836
Female	64	62	
**Tobacco smoking history**			
Stages 3-5	176	151	0.013*
Stages 1-2	61	86	
**Other malignancy history**			
Negative	214	215	0.884
Positive	27	26	
**Laterality**			
Left	111	94	0.100
Right	116	134	
**Location of lung parenchyma**			
Peripheral lung	48	43	0.503
Central lung	69	74	
**Residual tumor**			
Negative (R0)	191	195	0.445
Positive (R1/R2)	10	6	
**Tumor size**			
T1	46	62	0.081
T2-T4	196	180	
**Lymph node invasion**			
Negative	154	153	0.924
Positive	85	86	
**Distant metastasis**			
Negative	197	197	1.000
Positive	3	4	
**Tumor TNM stage**			
I/II	191	200	0.291
III/IV	49	40	

The clinical information from 484 LUSC patients was retrieved from cBioportal for the Cancer Genome Atlas (TCGA) databases for correlation analysis. LUSC: Lung squamous cell carcinoma. *$p<0.05$ was considered significant.

Figure S1

A

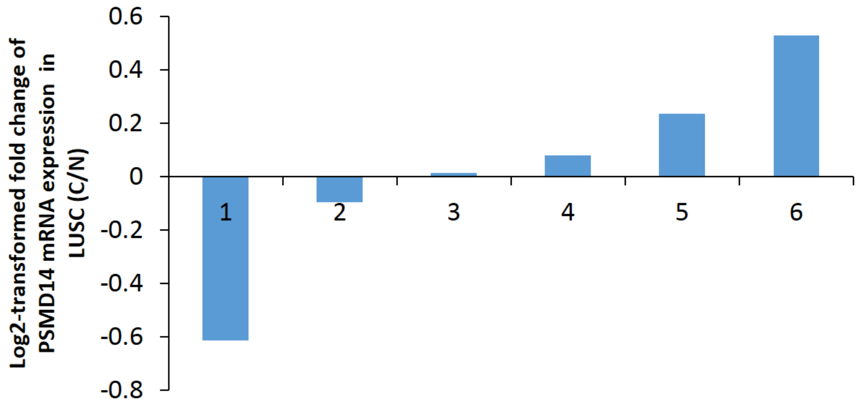

B

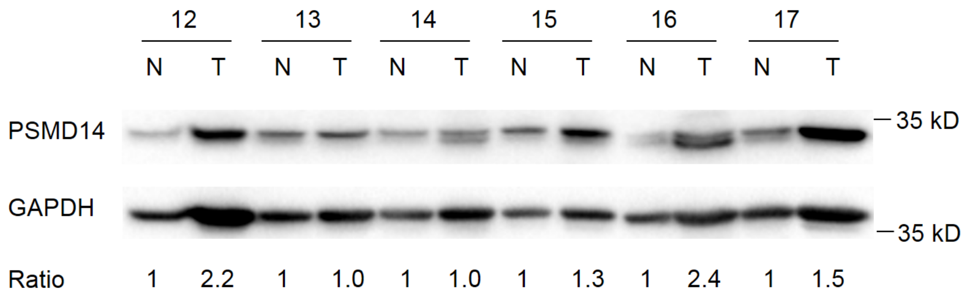

Figure S1 PSMD14 expression is upregulated in LUSC tissues. (A) PSMD14 mRNA expression in six pairs of LUSC tumor tissues and adjacent non-cancerous tissues was determined by RT-PCR. The ratio of PSMD14 expression in tumor tissues to controls for each patient is shown as a log2-transformed fold change. (B) PSMD14 protein expression in six pairs of tumor tissues and adjacent non-cancerous tissues in LUSC patients was determined by western blotting. PSMD14 expression was upregulated in four tumor samples and unchanged in two tumor samples. PSMD14 protein expression was elevated in LUSC tissues (p<0.05 by Fisher's exact test). LUSC, lung squamous cell carcinoma. T: tumor tissue; N: normal tissue.

Figure S2

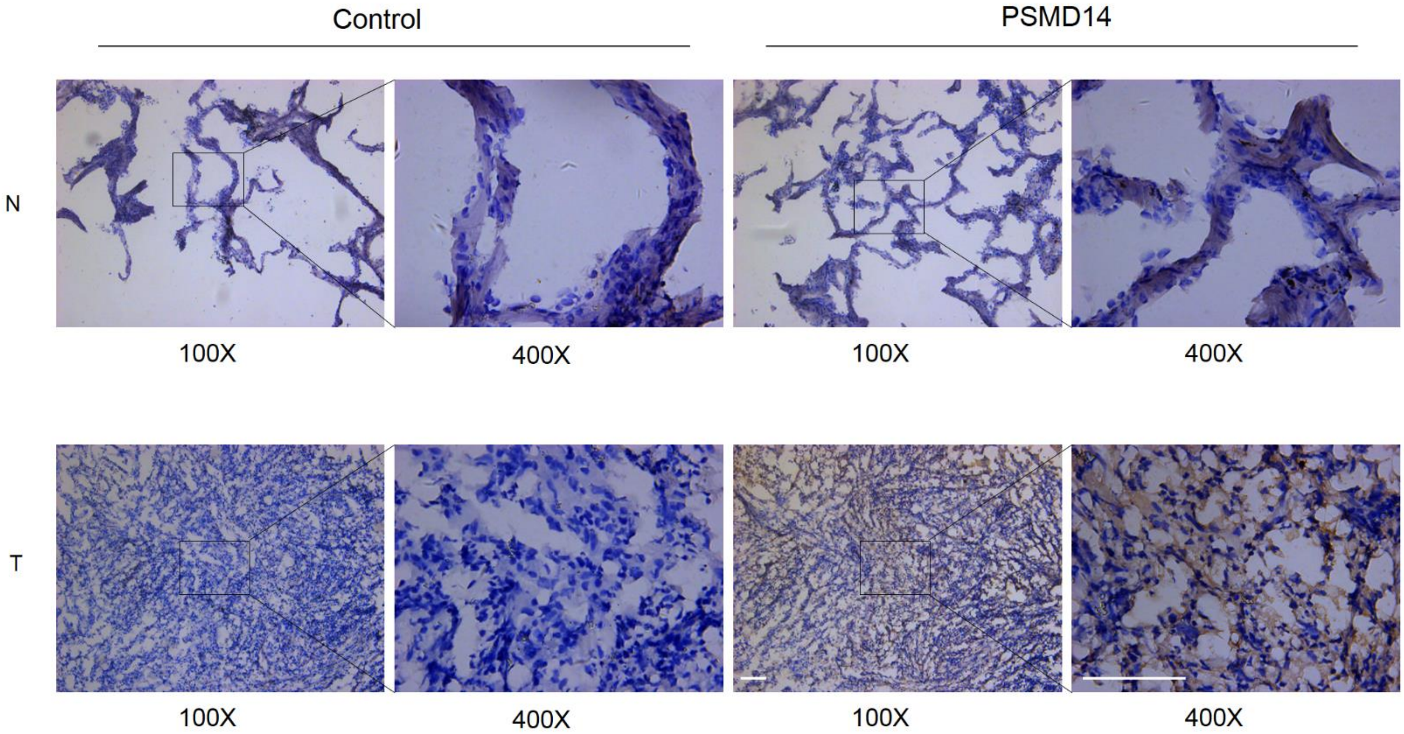

Figure S2 PSMD14 expression is upregulated in tumor tissues from LUAD patients. IHC staining of PSMD14 in tumor and paired adjacent non-cancerous tissues from LUAD patients was shown. The bar represents 100 μm. N: normal tissue; T: tumor tissue.

Figure S3

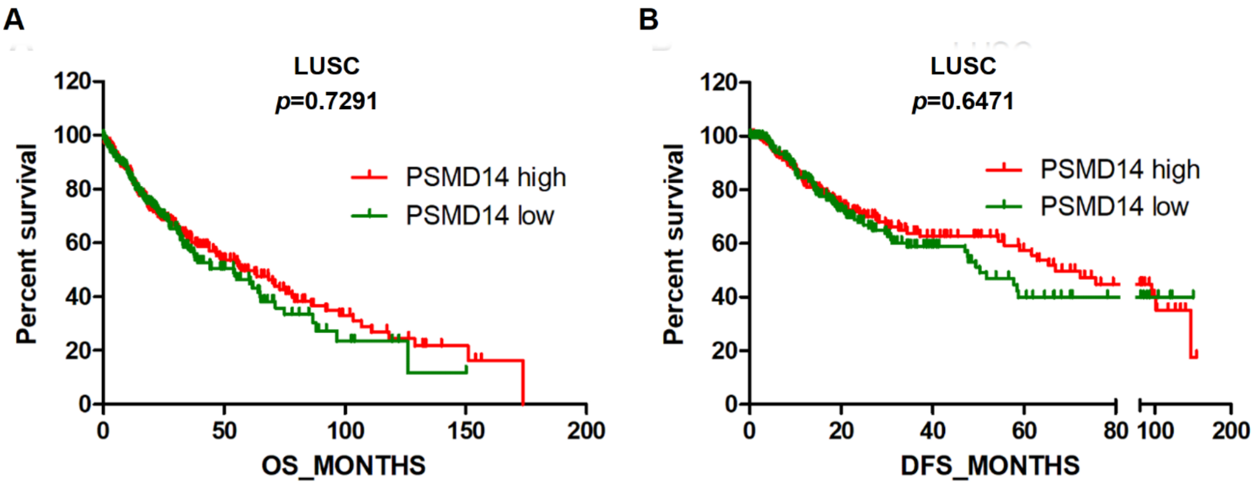

Figure S3 PSMD14 expression had no significant correlation with OS and DFS in LUSC patients. (A-B) Kaplan-Meier survival curves of higher and lower PSMD14 expression for OS (n=478) and DFS (n=364) in LUSC patients. The patients' information was retrieved from cBioportal for the Cancer Genome Atlasis (TCGA) databases and analyzed by a log-rank test. LUSC, lung squamous cell carcinoma; OS, overall survival; DFS, disease-free survival.

Figure S4

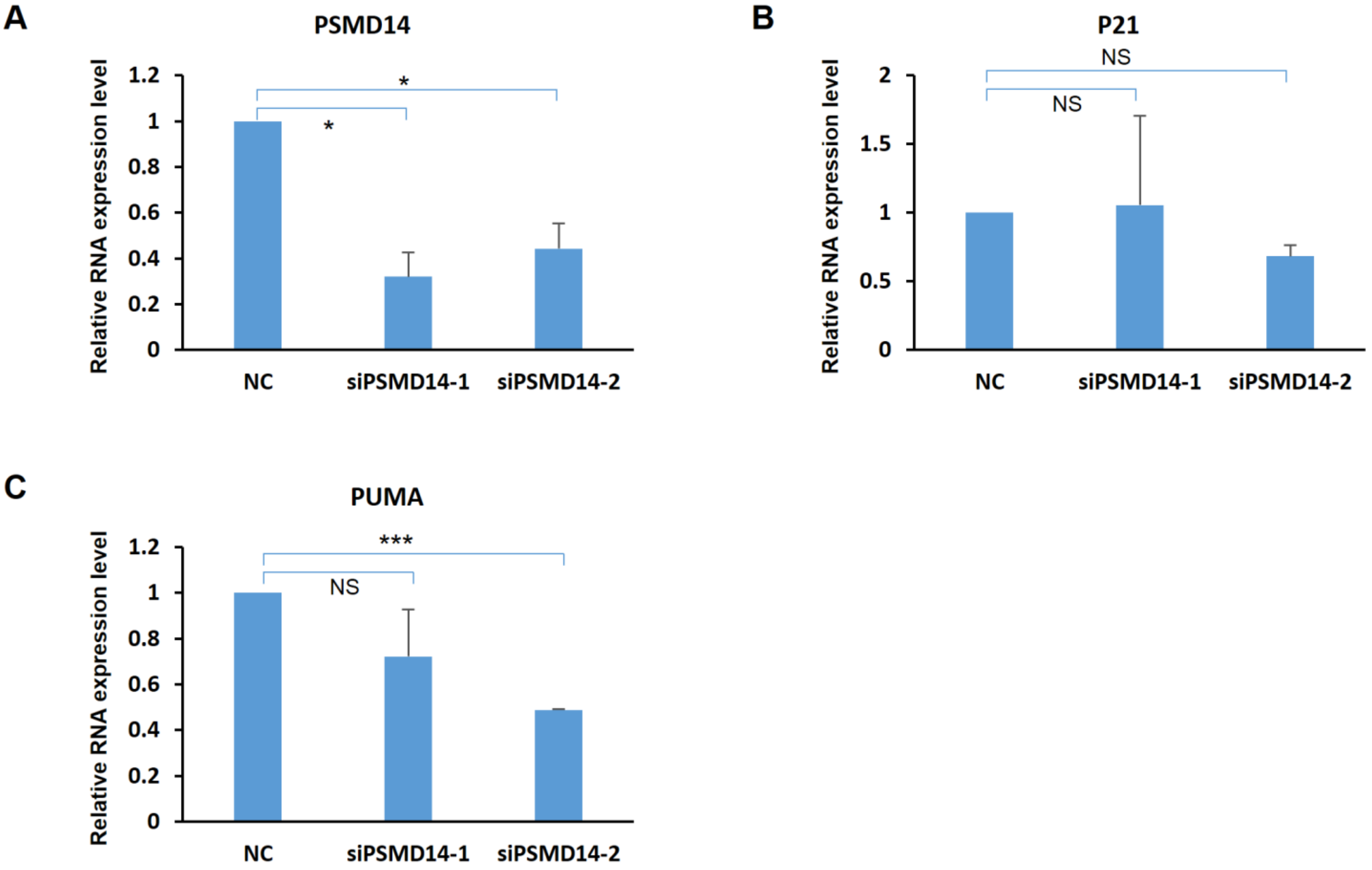

Figure S4 PSMD14 knockdown did not significantly increase the mRNA expression of p21 or PUMA. (A-C) RT-PCR results showing the relative mRNA expression of PSMD14, p21, and PUMA in A549 cells transfected with NC or si-PSMD14. *p<0.05 and ***p<0.001 were considered significant. NS was considered not significant.
